# Integrated DNA Methylation/RNA Profiling in Middle Temporal Gyrus of Alzheimer’s Disease

**DOI:** 10.1007/s10571-022-01307-3

**Published:** 2023-01-03

**Authors:** Ignazio S. Piras, Danielle Brokaw, Yinfei Kong, Daniel J. Weisenberger, Jonida Krate, Elaine Delvaux, Swapna Mahurkar, Adam Blattler, Kimberly D. Siegmund, Lucia Sue, Geidy E. Serrano, Thomas G. Beach, Peter W. Laird, Matthew J. Huentelman, Paul D. Coleman

**Affiliations:** 1 Neurogenomics Division, Translational Genomics Research Institute, Phoenix, AZ 85004 USA; 2 Biodesign Institute, Neurodegenerative Disease Research Center, Arizona State University, Tempe, AZ 85287 USA; 3 Department of Information Systems and Decision Sciences, California State University Fullerton, Fullerton, CA 92831 USA; 4 Department of Biochemistry and Molecular Biology, University of South California, Los Angeles, CA 90033 USA; 5 Biodesign Institute, Neurodegenerative Disease Research Center, Arizona State University, Tempe, AZ USA; 6 UCLA Division of Digestive Diseases, University of California, Los Angeles, CA 90024 USA; 7 Genetics Graduate Group, University of California, Davis, CA 95616 USA; 8 Department of Preventive Medicine, University of Southern California, Los Angeles, CA 90089-9175 USA; 9 Civin Laboratory of Neuropathology, Banner Sun Health Research Institute, Sun City, AZ 85351 USA; 10 Center for Epigenetics, Van Andel Institute, Grand Rapids, MI 49503 USA; 11 Present Address: UnityPoint Clinic, Waterloo, IA USA

**Keywords:** Alzheimer’s disease, DNA methylation, Epigenomics, RNA expression, Multi-omics

## Abstract

Alzheimer’s disease is a neurodegenerative disorder clinically defined by gradual cognitive impairment and alteration in executive function. We conducted an epigenome-wide association study (EWAS) of a clinically and neuropathologically characterized cohort of 296 brains, including Alzheimer’s disease (AD) and non-demented controls (ND), exploring the relationship with the RNA expression from matched donors. We detected 5246 CpGs and 832 regions differentially methylated, finding overlap with previous EWAS but also new associations. CpGs previously identified in *ANK1, MYOC,* and *RHBDF2* were differentially methylated, and one of our top hits (*GPR56*) was not previously detected. *ANK1* was differentially methylated at the region level, along with *APOE* and *RHBDF2*. Only a small number of genes showed a correlation between DNA methylation and RNA expression statistically significant. Multiblock partial least-squares discriminant analysis showed several CpG sites and RNAs discriminating AD and ND (AUC = 0.908) and strongly correlated with each other. Furthermore, the CpG site cg25038311 was negatively correlated with the expression of 22 genes. Finally, with the functional epigenetic module analysis, we identified a protein–protein network characterized by inverse RNA/DNA methylation correlation and enriched for “Regulation of insulin-like growth factor transport”, with *IGF1* as the hub gene. Our results confirm and extend the previous EWAS, providing new information about a brain region not previously explored in AD DNA methylation studies. The relationship between DNA methylation and gene expression is not significant for most of the genes in our sample, consistently with the complexities in the gene expression regulation.

**Graphical Abstract:**

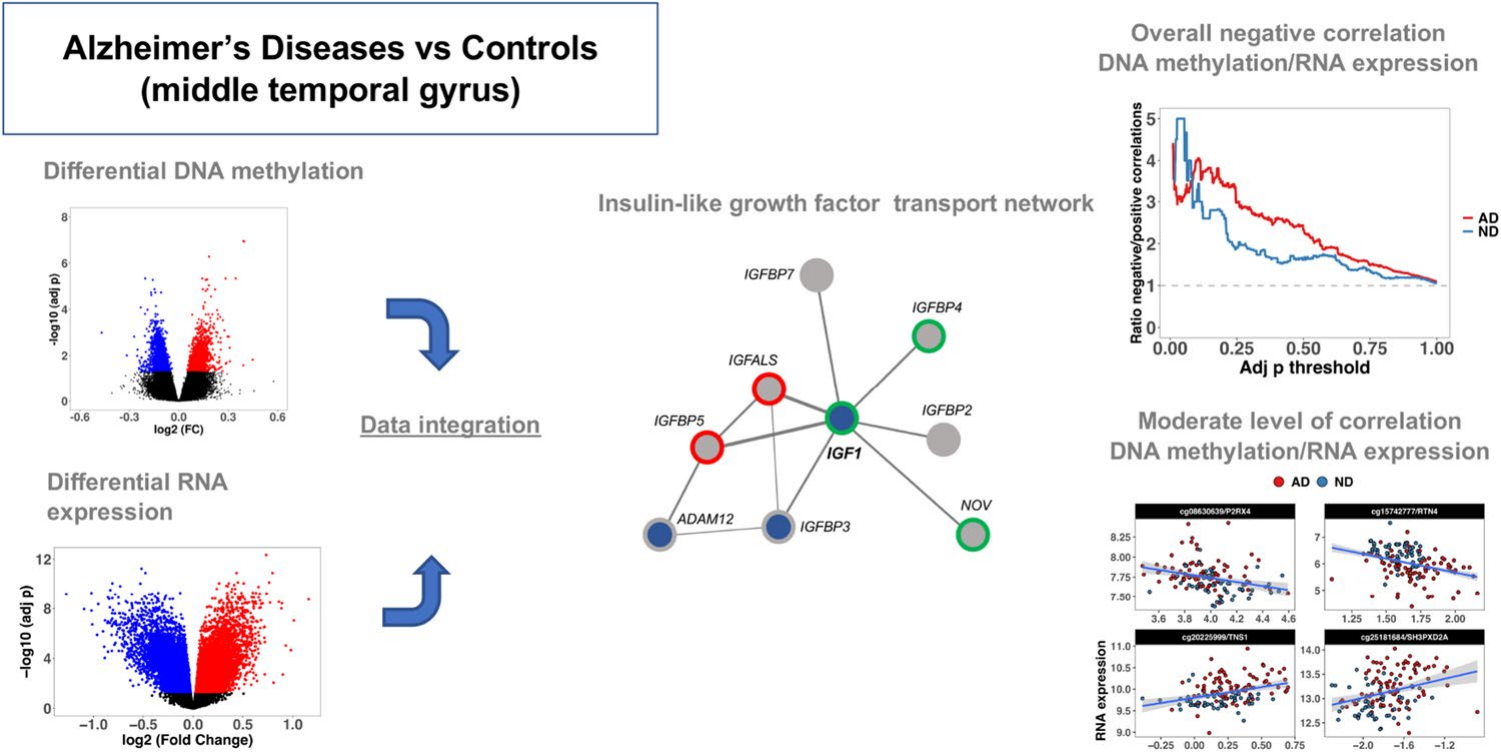

**Supplementary Information:**

The online version contains supplementary material available at 10.1007/s10571-022-01307-3.

## Background

Alzheimer’s disease is a neurodegenerative disorder clinically defined by gradual cognitive impairment and alteration in executive function. The symptoms are correlated to the loss of synaptic connections and neuronal cell death (Braak and Braak 1991; Dubois et al. 2010). The neuropathological hallmarks of AD are the accumulation of amyloid-β plaques (Aβ) and neurofibrillary tau tangles (NFTs) (Alzheimer’s Association 2015). AD is the most common cause of dementia in older adults, with affected people worldwide being currently estimated at 40 million and projected to double every 20 years until at least 2050 (Prince et al. 2015).

Many types of changes have been reported in the AD brain as synaptic and mitochondrial dysfunctions, impaired insulin signaling, vascular effects, inflammation, loss of calcium regulation, axonal transport deficits, aberrant cell-cycle reentry, and defects in the cholesterol metabolism (Querfurth and LaFerla 2010). At the genomic levels, AD brain RNA profiling has been deeply characterized using large cohorts across several brain regions by microarray or RNA sequencing (Guo et al. 2013; Lambert et al. 2013; Zhang et al. 2013; Allen et al. 2016; Wang et al. 2016, 2018; Mostafavi et al. 2018; Raj et al. 2018; Mathys et al. 2019; Piras et al. 2019a). In the last years, also the epigenomic landscape has been explored across multiple regions of the AD brain using DNA methylation microarrays (De Jager et al. 2014; Lunnon et al. 2014; Semick et al. 2019).

Aiming to investigate the relation between DNA methylation and gene expression and to extend and confirm findings from previous studies, here we used a sample of neuropathologically characterized 296 brains from AD and non-demented controls (ND) to investigate DNA methylation changes in middle temporal gyrus, a region not investigated in previous epigenome-wide association studies (EWAS). We also conducted an integrative multi-omics analysis using RNA profiling data from the same donors and the same tissue blocks from the same brain region.

### Methods

#### Sample Selection

Samples of the middle temporal gyrus (MTG, Brodmann area 21) were obtained from 296 brains (198 AD and 98 ND) from the Banner Sun Health Research Institute Brain and Body Donation Program (Beach et al. 2015; brain and body donation program.org). Samples had been frozen at autopsy and stored at − 80 °C. The cognitive status of all cases was evaluated ante-mortem by board-certified neurologists using Mini-Mental State Exam (MMSE), Global Deterioration Test (GDS), and Montreal Cognitive Assessment. These tests, along with general, medical, neurological, and movement examinations, are conducted annually on most subjects participating in the Brain and Body Donation Program. The diagnosis was made postmortem by a board-certified neuropathologist, resulting in a final consensus diagnosis using standard NIH AD Center criteria for AD or neurologically normal, non-demented elderly control (ND). Further details about the clinical assessment are reported in Beach et al. (2015). We included patients and controls with expired ages equal to or greater than 65 years old. All AD cases selected had a clinical diagnosis of dementia, Clinical Dementia Rating (CDR) scores ≥ 1, Braak Stage IV-VI, and Plaque Density “moderate” or “frequent”. ND samples were included in the study when matching the following criteria: CDR scores ≤ 0.5, Braak Stage 0–III, and Plaque Density “zero” or “sparse”. Braak Stage is a system for the assessment of AD-related neurofibrillary changes at the autopsy. AD-related neurofibrillary pathology is classified into six stages according to the spreading sites: entorhinal and transentorhinal cortex (stage I), hippocampus (stage II), temporal cortex (stage III), other regions of the cerebral cortex (stage IV), visual association cortex (stage V) and primary visual cortex (stage VI) (Braak et al. 2006).

#### DNA Extraction and Microarray Processing

Approximately 76 mg of frozen tissue was placed in a microfuge tube containing 0.5 ml lysis buffer (100 mM Tris–HCl pH 8.5, 5 mM EDTA, 0.2% SDS, 200 mM NaCl, and 100 μg/ml Proteinase K (Sigma)), freshly added and placed at 55 °C on a heat block overnight. After 1–2 h of incubation, a hand-held pellet pestle mixer (Kontes) was used to break up tissue. The next day, samples were further homogenized if needed. Four microliters of RNase A (Qiagen, 19,101) was added and set at room temperature (RT) for 30 min. After RNase treatment, equal volumes of phenol/chloroform/isoamyl alcohol (Sigma, P3803) were added. The tube was gently vortexed and then placed on a rocking platform for 5 min. After the samples were centrifuged at RT for 10 min at 10,000 RPM, the aqueous phase was transferred to a clean tube. Samples were ethanol-precipitated overnight at − 20 °C. Pellets were resuspended in 50 μl TE buffer (pH 8.0), quantified, checked for purity via spectrophotometry, and then stored at − 20 °C. Following bisulfite conversion, Infinium HumanMethylation450 (HM450) BeadChips were used to quantify DNA methylation of 404 samples from the middle temporal gyrus.

#### Data Processing

The preprocessing, quality controls and normalization were conducted using the *minfi* R-package (Fortin et al. 2017). Samples with more than 5% of probes with detection *p-*value ≥ 0.05 were excluded, as well as samples with an average detection *p-*value ≥ 0.05. A sex check was conducted using probes mapping to X and Y chromosomes. DNA methylation values were quantile-normalized using the *preprocessQuantile* function as implemented in *minfi*. We conducted a strict probe filtering, removing: probes located in single nucleotide polymorphisms (SNPs), probes with detection *p* ≥ 0.05 in at least one sample, probes located in sex chromosomes, and cross-reactive probes. The *M* values were used for all the downstream analyses (Du et al. 2010). We conducted multidimensional scaling (MDS) using the normalized filtered data aiming to detect batch effects and outliers. The relationship between DNA methylation, postmortem interval (PMI), and expired age was assessed using Pearson’s correlation. We estimated the cell-type composition (neuron positive and neuron negative proportions) using a deconvolution algorithm (Houseman et al. 2012) based on a dorsolateral prefrontal cortex (DLPFC) flow-sorted dataset (Guintivano et al. 2013). The difference in cell proportions between AD and ND was assessed using a linear regression (cell proportion as dependent variable and status as predictor, adjusting for age, sex, batch, PMI, and ethnicity) and a Wilcoxon test.

Differential methylation analysis between AD and ND was conducted at the single- and multi-probe (region) level. In both analyses, we modeled a linear regression using the *limma* R package (Ritchie et al. 2015), with DNA methylation as the dependent variable and AD/ND status as a predictor, adjusting for age, sex, cell proportions, batch, PMI, and ethnicity. Sex-specific differences were assessed by conducting the differential expression only in females and males and also using a two-way ANOVA model, as implemented in *limma.* The identification of differentially methylated regions (DMR) was conducted using the R-package *DMRcate* (Peters et al. 2015). *P*-values were adjusted using the false discovery rate (FDR) method (Benjamini and Hochberg 1995).

The relationship between DNA methylation and neuropathological variables (Braak Stage and Plaque Density) was investigated by modeling a linear regression (DNA methylation as dependent variables and the neuropathological variables as predictors), adjusting the model as conducted in the DNA methylation differential analysis. Both neuropathological variables were treated as continuous, the Braak stage ranging from 0 to 6, and the Plaque Density ranging from 0 to 4 (specifically: 0 = ”zero”, 1 = ”sparse”, 2 = ”moderate” and 3 = ”frequent”). For graphical representation purposes, we used the *M*-values adjusted for the covariates using the function *removeBatchEffect* as implemented in *limma.*

#### DNA Methylation Data Comparison with Prior Data

We downloaded the differential methylation results from 3 landmark studies investigating DNA methylation in AD brains across multiple regions (De Jager et al. 2014; Lunnon et al. 2014; Semick et al. 2019). Results from De Jager et al. (2014) included 71 CpG sites associated at the genome-wide level (*p* < 1.20E-07) with the burden of AD pathology in the dorsolateral prefrontal cortex. Results from Lunnon et al. (2014) included the top 100 CpG sites independently associated in four different brain regions (entorhinal cortex; cerebellum; prefrontal cortex; and superior frontal gyrus) and in a cross-cortical meta-analysis (*n* = 26 CpG sites). Finally, we used the 859 sites detected in the cross-region analysis and the 7253 in the region-dependent analysis filtered by FDR < 0.05 in the study by Semick et al. (2019).

#### Pathway Analysis

Enrichment analysis was conducted using the list of differentially methylated CpG sites, as well as the lists significantly correlated with the neuropathological variables. The analysis was conducted using the function *gometh* as implemented in the R-package *missMethyl* (Phipson et al. 2016)*,* taking into account the varying number of CpG sites associated with each gene and referencing to Gene Ontology (Ashburner et al. 2000) and KEGG (Kanehisa and Goto 2000) databases. We used the entire list of probes included in the differential methylation analysis as a background list. We conducted an additional analysis using the C2 “curated” and H “hallmark” genesets from the Broad Institute Molecular signature database (Subramanian et al. 2005). This last analysis was conducted using the function *gsameth* using the same settings as in the GO and KEGG analysis.

#### Multi-Omics Analysis with Matched RNA Expression Profiles

We leveraged a dataset including RNA expression profiling from the same donors and the same brain region of the samples used in the current study for a total of 135 overlapping samples (82 AD and 53 ND). Sample description and RNA profiling characterization are reported in our previous study (Piras et al. 2019a).

We followed four different approaches for the RNA/DNA methylation integration: (1) correlation approach to investigate the general relationship between RNA profiling and DNA methylation and how this relationship possibly changes by the disease status; (2) overlap of significant DMRs and differentially expressed genes (DEGs) to detect genes simultaneously differentially expressed and methylated; (3) Data Integration Analysis for Biomarker discovery using Latent Component (DIABLO), with the goal to identify correlated features also associated with the disease status; (4) Functional Epigenetic Module analysis, aiming to detect interactome hotspots differentially expressed and methylated (FEM).

The first approach (general relationship between DNA methylation and gene expression) was carried out by running a bi-weighted correlation (Langfelder and Horvath 2008) between RNA expression levels and CpG sites mapping ± 1500 bp from the corresponding gene. The *R*^*2*^ was computed using the R function *lmrob*, computing the fast MM-type estimator. P-values were adjusted for multiple testing using the FDR method.

The second approach (overlap of significant DMRs and DEGs) was conducted computing differential RNA expression between AD and ND using the 135 overlapping samples by means of a linear model as implemented in *limma* and adjusting for age, sex, PMI, and RIN. The differential DNA methylation analysis at the gene level was conducted for the 135 overlapping DNA methylation samples as well. Then, we intersected the results matching the significant differentially methylated probes (DMPs) and DEGs (adj *p* < 0.05) by ensemble gene ID.

The third type of multi-omics analysis (DIABLO) was carried out using the R-package *mixOmics* (Rohart et al. 2017). DIABLO is a sparse generalized canonical correlation discriminant analysis generating a linear combination of the original data (here, DNA methylation and RNA expression profiles), allowing the identification of molecular features jointly associated with the response variable (here, the disease status). We used the same input data used in the previous analytical approach (adjusted matrix of M and expression values), including only the top 50% genes (RNA data) and CpG sites (DNA methylation data) with the highest median absolute deviation (MAD). The optimal model of hyperparameters (the number of components and variables) was determined using cross-validation (20 × fivefold) using the functions *tune.splsda* and *perf.* The final model was run using the function *block.splsda*, and the area under the curve (AUC) was computed using the *auroc* function.

Finally, the last analytical method consisted of functional epigenetic module analysis (FEM) analysis, capable of identifying interactome hotspots of differential promoter DNA methylation and expression inverse correlated (Jiao et al. 2014) and based on protein–protein interactions (PPI). The sub-networks are selected from the rest of the network when they have an exceptionally large average edge-weight density, where the weight edges are generated according to the association effect size with the phenotype of interest (here, the disease status). The method is implemented in the *FEM* R package (Jiao et al. 2014). As input, we used the adjusted M and RNA expression value matrices. Since the RNA expression data included multiple probes mapping to the same genes, we included those with the highest average expression. The first step of the analysis for both RNA and DNA methylation data consists of the generation of summary differential analysis statistics (AD vs ND) using the *GenStatM* and *GeneStatR* functions, respectively. Then, we created a PPI adjacency matrix from *Biogrid v3.5.180* database (Oughtred et al. 2019) to integrate with the summary differential expression and methylation statistics. Finally, the identification of interactome hotspots of differential promoter methylation and differential expression in relation to a phenotype of interest was conducted using the function *DoFEMbi* with: *nseeds* = 100, *gamma* = 0.5, *nMC* = 10,000, selecting a range of modules including from 5 to 100 nodes.

### Results

#### Quality Controls

The DNA methylation profiling was conducted on 296 samples (198 AD and 98 ND). We did not detect any sex mismatch, and six samples were excluded because showing more than 5% of probes with a detection *p*-value ≥ 0.05. The remaining samples showed an average detection *p* < 0.05 (AD = 194; ND = 96). After quantile normalization (density plots shown in Supplementary Fig. 1), we conducted probe filtering starting from all the available 485,512 probes. We removed SNPs mapping probes (*n* = 17,541), probes with detection *p* ≥ 0.05 in at least one sample (*n* = 52,543), probes located in sex chromosomes (*n* = 8618), and cross-reactive probes (*n* = 24,656). The final number of probes after filtering was 381,974 (78.7% of the initial number). We conducted MDS analysis using the top 2 components, plotting the samples by status and batch (Supplementary Fig. 2). We detected a significant correlation of the top 2 principal components with the plate through multinomial regression (PC1: lowest *p* = 1.1E-13; PC2: lowest *p* = 2.2E-16), but no significant correlation with age, sex, or PMI (Spearman’s correlation and Wilcoxon test: *p* ≥ 0.278). The batch effect was removed after regressing out the confounding factors (PC1 and PC2: lowest *p* = 1.000) (Supplementary Fig. 3B).

The final dataset used for the downstream analyses was composed of 194 AD and 96 ND (Table 1). The average age for the AD group was larger but not significantly different from the ND group (*p* = 0.071) (Supplementary Fig. 4A). Sex distribution was significantly different between AD and ND (*p* = 4.4E-04) (Supplementary Fig. 4B), but not the PMI (*p* = 0.247) (Supplementary Fig. 5).

**Table 1:** Demographic characteristics of the samples passing quality controls

Variable	AD (*n* = 194)	ND (*n* = 96)	*p*
Age	83.8 ± 6.4 (65–102)	82.2 ± 7.4 (65–102)	0.071
Sex (M:F ratio)	0.8	2.0	4.4E-04

Mean ± standard deviation, and range are reported

Among AD, most of the participants showed Braak Stage V and IV (46.4% and 39.7%, respectively), whereas 40.6% of ND showed Braak Stage III. AD patients had a large prevalence of “frequent” Plaque density (82.0%), whereas the prevalence of “zero” and “sparse” in ND was comparable (53.7% and 46.3%, respectively) (Supplementary Fig. 6). Finally, brain weight and MMSE were significantly lower in AD (Supplementary Fig. 7). We estimated the cell proportions between AD and ND, finding a significant lower neuronal prevalence in AD with both linear regression (*β* =  − 0.037 ± 0.009; *p* = 3.2E-05) and Wilcoxon test (*W* = 6509; *p* = 3.0E-05) (Supplementary Fig. 8).

#### Differential Methylation Between AD and ND at the CpG Site Level

**Fig. 1:**
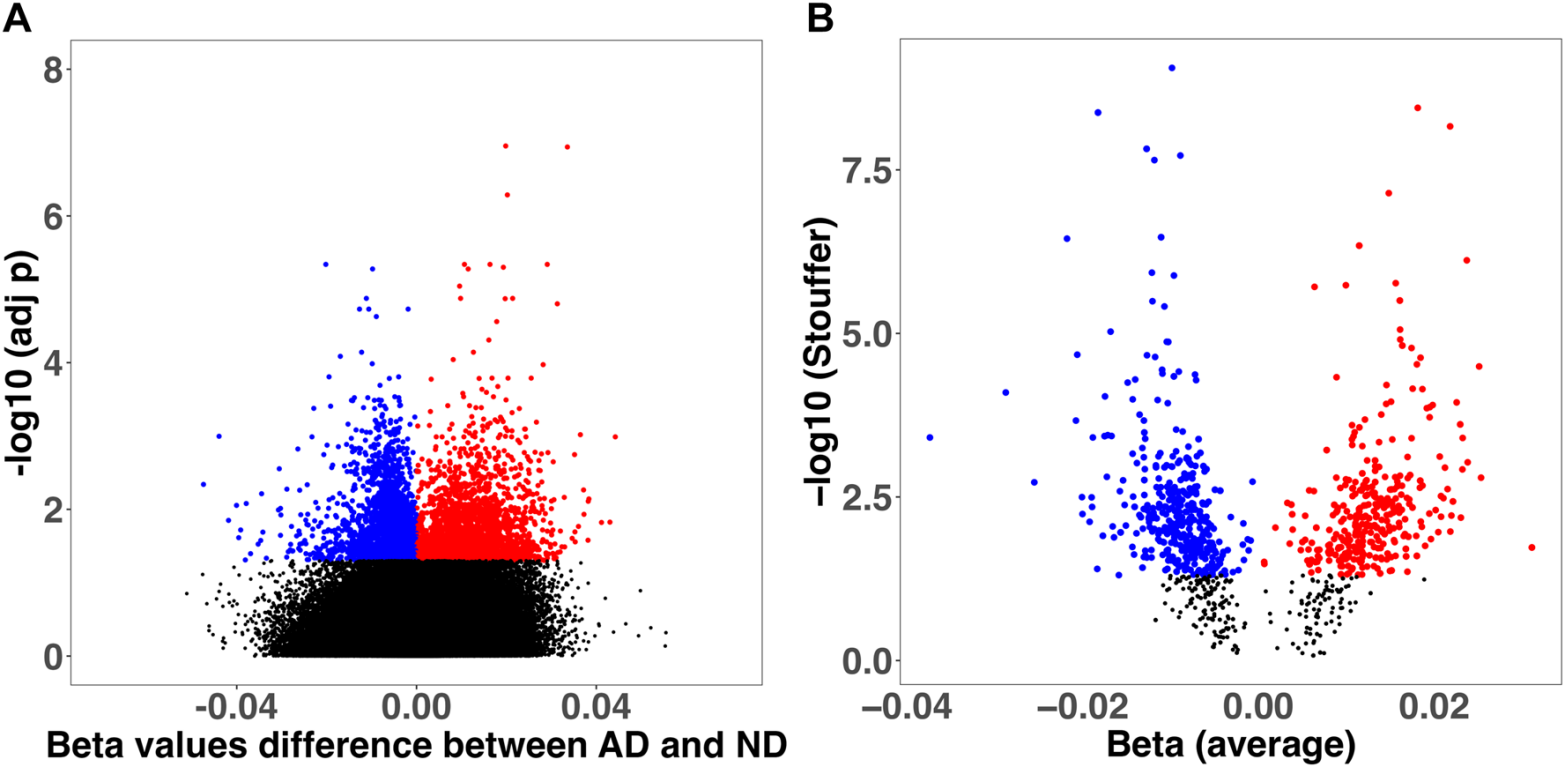
Volcano plots showing the differential methylation results at the probe (**A**) and region (**B**) level Effect sizes (x-axis) are shown as differences in beta values between groups

We obtained 5246 differentially methylated sites (FDR < 0.05), 2657 hypermethylated and 2589 hypomethylated (Fig. 1a). However, only the 34.2% of CpG sites had a 1% or larger variation in β-values (Δβ) between AD and ND. The median Δβ for the significantly methylated CpGs was 0.7%, with the largest value of 4.7%. The most prominent associations by adj-*p* and effect size were two hypermethylated CpG sites located in the *ANK1* gene (cg11823178) and in the promoter of the *GPR56* gene. The top 20 CpG sites ranked by *p*-values are reported in Table 2*,* and the complete significant results are reported in Supplementary Table 1.

**Table 2:** Top 20 CpG sites differentially methylated between AD and ND

Chr	Position	Name	Islands name	Relationship to gene	Symbol	Δβ	Average (M values)	t	*p*	Adj *p*
8	41,519,399	cg11823178		Body; TSS1500	*ANK1; MIR486*	0.020	3.703	7.667	2.9E-13	1.1E-07
8	41,519,308	cg05066959		Body; TSS1500	*ANK1; MIR486*	0.034	2.709	7.554	6.0E-13	1.1E-07
8	119,122,430	cg05313153	chr8:119,123,974–119,124,432	1st Exon	*EXT1*	0.020	− 2.245	7.251	4.1E-12	5.2E-07
8	42,033,472	cg17693222		3’UTR	*PLAT*	0.011	2.580	6.838	5.0E-11	4.6E-06
3	188,664,632	cg12307200	chr3:188,665,275–188,665,552			− 0.020	2.480	− 6.786	6.9E-11	4.6E-06
16	57,673,258	cg09109520		5’UTR	*GPR56*	0.029	− 2.206	6.778	7.2E-11	4.6E-06
17	1,373,605	cg05417607	chr17:1,375,216–1,375,460	Body	*MYO1C*	0.016	3.592	6.752	8.4E-11	4.6E-06
10	22,603,999	cg10713515	chr10:22,604,907–22,605,632	TSS1500	*COMMD3*	0.019	0.907	6.715	1.0E-10	5.0E-06
7	218,121	cg23782833	chr7:218,309–218,951	Body	*FAM20C*	− 0.010	2.189	− 6.678	1.3E-10	5.3E-06
3	86,703,014	cg14526375				0.011	1.461	6.668	1.4E-10	5.3E-06
17	74,475,294	cg13076843	chr17:74,475,239–74,475,452	Body	*RHBDF2*	0.010	3.429	6.559	2.6E-10	9.1E-06
17	72,779,428	cg22312249		Body	*TMEM104*	0.021	2.520	6.476	4.2E-10	1.3E-05
2	75,005,912	cg08308032				0.010	− 0.301	6.461	4.6E-10	1.3E-05
5	1,033,518	cg15165927		Body	*NKD2*	− 0.011	1.782	− 6.451	4.9E-10	1.3E-05
21	47,855,893	cg23449541	ch*r2*1:47,855,892–47,856,143	Body	*PCNT*	0.020	2.836	6.437	5.3E-10	1.3E-05
15	74,836,094	cg19144497	chr15:74,833,241–74,833,961	5’UTR	*ARID3B*	0.031	0.731	6.398	6.6E-10	1.6E-05
22	20,236,751	cg08753951		TSS200; Body	*MIR1286; RTN4R*	− 0.002	1.158	− 6.352	8.6E-10	1.9E-05
4	159,092,536	cg03304437		5’UTR	*FAM198B*	− 0.013	0.581	− 6.344	9.0E-10	1.9E-05
11	94,600,954	cg26130242		Body	*AMOTL1*	− 0.011	1.976	− 6.338	9.3E-10	1.9E-05
5	173,180,686	cg23975425				− 0.009	2.551	− 6.288	1.2E-09	2.4E-05

All CpG sites listed but one have a change in DNA methylation equal to or larger than 1%

We ran a sensitivity analysis without adjusting for cell type, detecting a total of 32,965 significant DMPs, with 2648 of them significant in both the cell-adjusted and non-cell-adjusted analyses. The correlation of the test statistics (log2 FC) between the two analyses was strong and statistically significant (*p* = 0.654, *p* < 2.2E-16). The distribution of significantly methylated CpG sites according to the genomic density features was statistically different between significant and non-significant (Chi-square test: *p* < 2.2E-16) sites. Among the differentially DNA methylated sites, we noticed a decrease in CpG sites in the islands and an increase in open sea and shelves (all *p* < 2.2E-16) , but not in shore regions (*p* = 0.757). (Supplementary Fig. 9A). We investigated the CpGs distribution according to their location relative to the gene, considering 3’UTR, TSS1500, TSS200, 1st Exon, Body, and 5’UTR. Overall, we found a significantly different distribution between significant and non-significant CpGs (*p* < 2.2E-16). Additionally, the distribution was significantly different for each gene location, with p-values ranging from 9.7E-03 (5’UTR) to gene body (*p* < 2.2E-16) (Supplementary Table 2; Supplementary Fig. 9B).

We conducted differential methylation analysis also on males and females separately, detecting 63 significant CpG sites in males and 94 sites significant in females, with an overlap of only three sites differentially methylated between the two analyses. Two-way ANOVA did not detect any CpG sites significantly differentially methylated only in females or males. Finally, most of the CpG sites detected in males and females were differentially methylated in the total (males + females) analysis, with 92.1% and 89.4% of sites in males and females, respectively (Supplementary Table 3).

We compared our significant results with eight different analyses from 3 large EWAS. We detected a total of 359 sites validated at least in 1 comparison (Supplementary Table 4), with 14 of them validated in at least 4 (50%) comparisons (Supplementary Fig. 10). Specifically, three sites were validated in 6 (75%) of comparisons: cg11823178 (*ANK1*), cg05417607 (*MYOC1*), and cg05810363 (*RHBDF2)*. Two additional sites were instead validated in five (62.5%) studies located in *ANK1* and *SPG7.* Finally, nine CpG sites were validated in four (50%) datasets. These sites were located in: *RHBDF2* (*n* = 3), *PCNT* (*n* = 2), *CHD23/C10orf54*, *ALDH16A1*, *ABR* and *ZZEF1* (all with one CpG site). On the other hand, 4887 CpG sites detected differentially methylated in our study were not cross-validated in these datasets and can be considered new associations (Supplementary Table 5). The top CpG site non-validated ranked by adj *p* was located in the *GPR56* gene (cg09109520), whereas other top sites were located in *COMMD3*, *FAM20C*, *TMEM104,* and *NKD2* (all: adj *p* < 1.0E-04). The % validation was not significantly correlated with the *Δβ* (*p* = 0.083; *p* = 0.116).

The pathways analysis was conducted using different methods and databases, accounting for the proportion of probes/genes. After multiple test correction and using all the DMPs, we obtained only significant enriched processes for the Hallmark gene sets (Estrogen response early) (Supplementary Table 6). We repeated the analysis by separating hypermethylated and hypomethylated probes and also considering the location of the CpGs related to the gene (including 3’UTR, TSS1500, TSS200, 1^st^ Exon, Body, and 5’UTR) not finding significant results after multiple test adjustment (data not shown).

#### Differential Methylation Between AD and ND at the Region Level

We detected 832 DMRs (393 hypermethylated and 439 with hypomethylated trend) (Fig. 1b). The most significant associations were located in the genes: *PRDM16*, *MCF2L*, *FAM198B*, and *GCNT2 (*adj *p* < 1.0E-08)*.* Interestingly, a significant region encompassing *ANK1* was significantly hypermethylated, ranking among the top 20 regions (Table 3).

**Table 3:** Top 20 differentially methylated regions between AD and ND

Chr	Start	End	Symbol	Width (bp)	CpGs	Mean beta fc	Stouffer
1	3,191,219	3,193,280	*PRDM16*	2062	11	− 0.010	8.7E-10
13	113,698,408	113,700,027	*MCF2L*	1620	16	0.018	3.6E-09
4	159,091,837	159,092,553	*FAM198B*	717	4	− 0.018	4.2E-09
6	10,555,808	10,556,523	*GCNT2*	716	5	0.022	6.8E-09
2	27,529,325	27,531,535	*TRIM54, UCN*	2211	18	− 0.013	1.5E-08
7	1,490,631	1,493,153	*MICALL2*	2523	12	− 0.009	1.9E-08
17	75,462,916	75,463,984	*SEPT9*	1069	5	− 0.012	2.2E-08
12	52,403,511	52,404,422	*GRASP*	912	6	0.015	7.2E-08
19	1,465,207	1,468,943	*APC2, C19orf25*	3737	16	− 0.011	3.4E-07
1	167,090,618	167,091,161	*DUSP27*	544	7	− 0.022	3.6E-07
8	41,519,026	41,519,399	*ANK1*	374	4	0.011	4.6E-07
3	121,612,956	121,613,396	*SLC15A2*	441	3	0.024	7.6E-07
4	2,065,749	2,066,397	*NAT8L*	649	5	− 0.012	1.2E-06
1	17,996,881	17,998,265	*ARHGEF10L*	1385	6	− 0.010	1.3E-06
6	130,182,053	130,182,967	*TMEM244*	915	8	0.016	1.7E-06
8	42,033,472	42,033,498	*PLAT*	27	2	0.010	1.8E-06
17	74,475,050	74,475,402	*RHBDF2*	353	6	0.006	1.9E-06
7	27,145,972	27,148,002	*HOXA3, HOXA-AS2*	2031	10	0.016	3.1E-06
8	143,442,626	143,442,764	*TSNARE1*	139	2	− 0.012	3.2E-06
19	45,411,802	45,412,647	*APOE*	846	4	− 0.011	3.9E-06

*APOE* gene was hypomethylated and ranked 20th. Full details on the 832 significant regions are reported in Supplementary Table 7. We conducted pathway analysis using REACTOME, but we did not detect any significance after multiple test adjustment. In the hypermethylated regions, the top class was “Transmission across chemical synapses” (*p* = 0.009), and in the hypomethylated genes, the top class was “Cell cycle–mitotic” (*p* = 0.025) (Supplementary Table 8).

#### Correlation with Neuropathological Variables: Braak Stage and Plaque Density

We tested the CpG sites associated with AD (*n* = 5246) to investigate the relationship of DNA methylation with Braak Stage and tangle density. We detected a total of 4025 CpG sites associated with the Braak stage, representing 76.7% of the DMPs analyzed. The correlation between the *β* coefficient of the linear regression and the log2 FC from the differential analysis was very strong and statistically significant (*r* = 0.904; *p* < 2.2E-16). The complete list of the CpG sites associated is reported in Supplementary Table 10, and the top 20 sites are reported in Table 4. Nine sites in Table 3 are also ranked in the top 20 CpG sites differentially methylated (i.e.: *ANK1*, *MYOC1*, *EXT1*, *PCNT*). However, some of the associated sites were not ranked in the top positions (i.e., CpG sites in *SLC13A5*, *MKNK1*, *A2M,* and *DUSP5).* The median effect size, computed as the difference between donors with Braak Stage VI and donors with Braak Stage I, was *Δβ* = 1.2% (maximum: 8.0%).

**Table 4:** Top CpG sites associated with the Braak stage

Chr	Pos	Probe	Islands name	Relationship to gene	Symbol	Beta (regression)	Δβ	*p*	BH	Rank (diff. analysis)
8	41,519,399	cg11823178		Body; TSS1500	*ANK1; MIR486*	0.124	0.031	2.2E-16	1.2E-12	1
8	41,519,308	cg05066959		Body; TSS1500	*ANK1; MIR486*	0.125	0.046	8.4E-16	2.2E-12	2
17	1,373,605	cg05417607	chr17:1,375,216–1,375,460	Body	*MYO1C*	0.103	0.023	3.1E-15	5.4E-12	7
3	188,664,632	cg12307200	chr3:188,665,275–188,665,552			− 0.068	− 0.024	9.6E-14	1.3E-10	5
8	119,122,430	cg05313153	chr8:119,123,974–119,124,432	1^st^Exon	*EXT1*	0.054	0.023	4.8E-13	5.1E-10	3
21	47,855,893	cg23449541	ch*r2*1:47,855,892–47,856,143	Body	*PCNT*	0.069	0.025	4.7E-12	4.0E-09	15
15	74,836,094	cg19144497	chr15:74,833,241–74,833,961	5’UTR	*ARID3B*	0.056	0.033	5.9E-12	4.0E-09	16
6	106,041,074	cg23316191				0.047	0.022	6.1E-12	4.0E-09	429
10	77,188,318	cg18456331	chr10:77,191,061–77,191,571			0.080	0.021	2.4E-11	1.4E-08	145
17	6,616,653	cg16652063	chr17:6,616,422–6,617,471	5’UTR	*SLC13A5*	− 0.044	− 0.018	5.6E-11	3.0E-08	166
8	42,033,472	cg17693222		3’UTR	*PLAT*	0.039	0.013	8.8E-11	3.6E-08	4
1	47,066,498	cg23277659	chr1:47,069,615–47,070,179	Body	*MKNK1*	0.073	0.027	8.8E-11	3.6E-08	425
17	32,741,121	cg20942162				0.051	0.020	9.3E-11	3.6E-08	78
3	139,557,385	cg20865082				0.060	0.023	9.9E-11	3.6E-08	118
10	22,603,999	cg10713515	chr10:22,604,907–22,605,632	TSS1500	*COMMD3*	0.048	0.012	1.0E-10	3.6E-08	8
13	40,396,777	cg16042652				0.070	0.027	1.1E-10	3.6E-08	144
12	9,265,989	cg08300930		Body	*A2M*	0.042	0.022	1.2E-10	3.8E-08	184
10	73,472,315	cg07571519		3’UTR; Body	*C10orf105; CDH23*	0.048	0.019	1.4E-10	4.0E-08	22
10	112,260,509	cg02927679	chr10:112,257,163–112,258,684	Body	*DUSP5*	0.060	0.023	1.4E-10	4.0E-08	252
5	15,993,627	cg25579729				0.063	0.019	1.6E-10	4.1E-08	3

We conducted the same analysis using the plaque density, detecting 3370 CpG sites significantly associated, representing 64.2% of the DMPs analyzed. The correlation between the coefficient *β* of the linear regression and the log2 FC from the differential analysis was very strong and significant (*r* = 0.836; *p* < 2.2E-16). The complete list of the CpG sites associated is reported in Supplementary Table 11, and the top 20 sites are reported in Table 5. A total of 10 sites in Table 4 are ranked in the top 20 in the differential methylation analysis (i.e., *ANK1*, *MYOC1*, *TMEM104,* and *ARID3B*)*.* However, as observed for the Braak stage, several sites are not ranked in the top positions (i.e., *DUSP5*, *CAPN3*, *ATXN1,* and *HMGN3*). The median effect size, computed as the difference between donors with “frequent” plaques and donors with “zero” plaques, was *Δβ* = 1.0% (maximum: 5.8%).

**Table 5:** Top CpG sites associated with the plaque density

Chr	Pos	Probe	Islands_name	Relationship to gene	Symbol	Beta (regression)	Δβ	p	BH	Rank (diff. analysis)
8	41,519,399	cg11823178		Body; TSS1500	*ANK1; MIR486*	0.168	0.023	6.7E-16	2.3E-12	1
8	41,519,308	cg05066959		Body; TSS1500	*ANK1; MIR486*	0.172	0.039	8.7E-16	2.3E-12	2
17	1,373,605	cg05417607	chr17:1,375,216–1,375,460	Body	*MYO1C*	0.141	0.020	2.6E-15	4.6E-12	7
17	72,779,428	cg22312249		Body	*TMEM104*	0.097	0.026	5.5E-15	6.3E-12	12
15	74,836,094	cg19144497	chr15:74,833,241–74,833,961	5’UTR	*ARID3B*	0.086	0.038	6.0E-15	6.3E-12	16
8	119,122,430	cg05313153	chr8:119,123,974–119,124,432	1stExon	*EXT1*	0.078	0.021	2.0E-14	1.8E-11	3
3	182,968,758	cg14761246	chr3:182,971,429–182,972,635	Body	*MCF2L2*	0.122	0.022	6.8E-14	5.1E-11	40
3	188,664,632	cg12307200	chr3:188,665,275–188,665,552			− 0.091	− 0.021	5.3E-13	3.5E-10	5
10	73,472,315	cg07571519		3’UTR; Body	*C10orf105; CDH23*	0.073	0.020	7.6E-13	4.4E-10	22
10	77,188,318	cg18456331	chr10:77,191,061–77,191,571			0.117	0.027	9.2E-13	4.8E-10	146
10	112,260,509	cg02927679	chr10:112,257,163–112,258,684	Body	*DUSP5*	0.090	0.029	1.4E-12	6.7E-10	253
3	36,782,467	cg21113478		TSS1500	*DCLK3*	0.090	0.027	2.8E-12	1.2E-09	242
12	9,265,989	cg08300930		Body	*A2M*	0.061	0.018	7.3E-12	2.9E-09	185
1	65,531,864	cg13390284	chr1:65,532,161–65,533,212			0.105	0.038	1.2E-11	4.5E-09	131
13	40,396,777	cg16042652				0.101	0.028	1.5E-11	4.9E-09	145
15	42,652,294	cg14833933		1stExon	*CAPN3*	0.074	0.030	1.5E-11	4.9E-09	576
6	16,758,889	cg00464814	chr6:16,760,178–16,762,985	5’UTR	*ATXN1*	0.095	0.023	2.4E-11	7.2E-09	738
10	22,603,999	cg10713515	chr10:22,604,907–22,605,632	TSS1500	*COMMD3*	0.068	0.019	2.5E-11	7.2E-09	8
17	32,741,121	cg20942162				0.071	0.026	3.1E-11	8.6E-09	78
21	47,855,893	cg23449541	ch*r2*1:47,855,892–47,856,143	Body	*PCNT*	0.092	0.021	3.3E-11	8.6E-09	15
8	42,033,472	cg17693222		3’UTR	*PLAT*	0.054	0.013	3.4E-11	8.6E-09	4
6	79,911,416	cg22032366		Body	*HMGN3*	0.107	0.032	4.0E-11	9.6E-09	329

### Relationship with Gene Expression

#### Overall Relationship DNA Methylation/RNA Expression

**Fig. 2:**
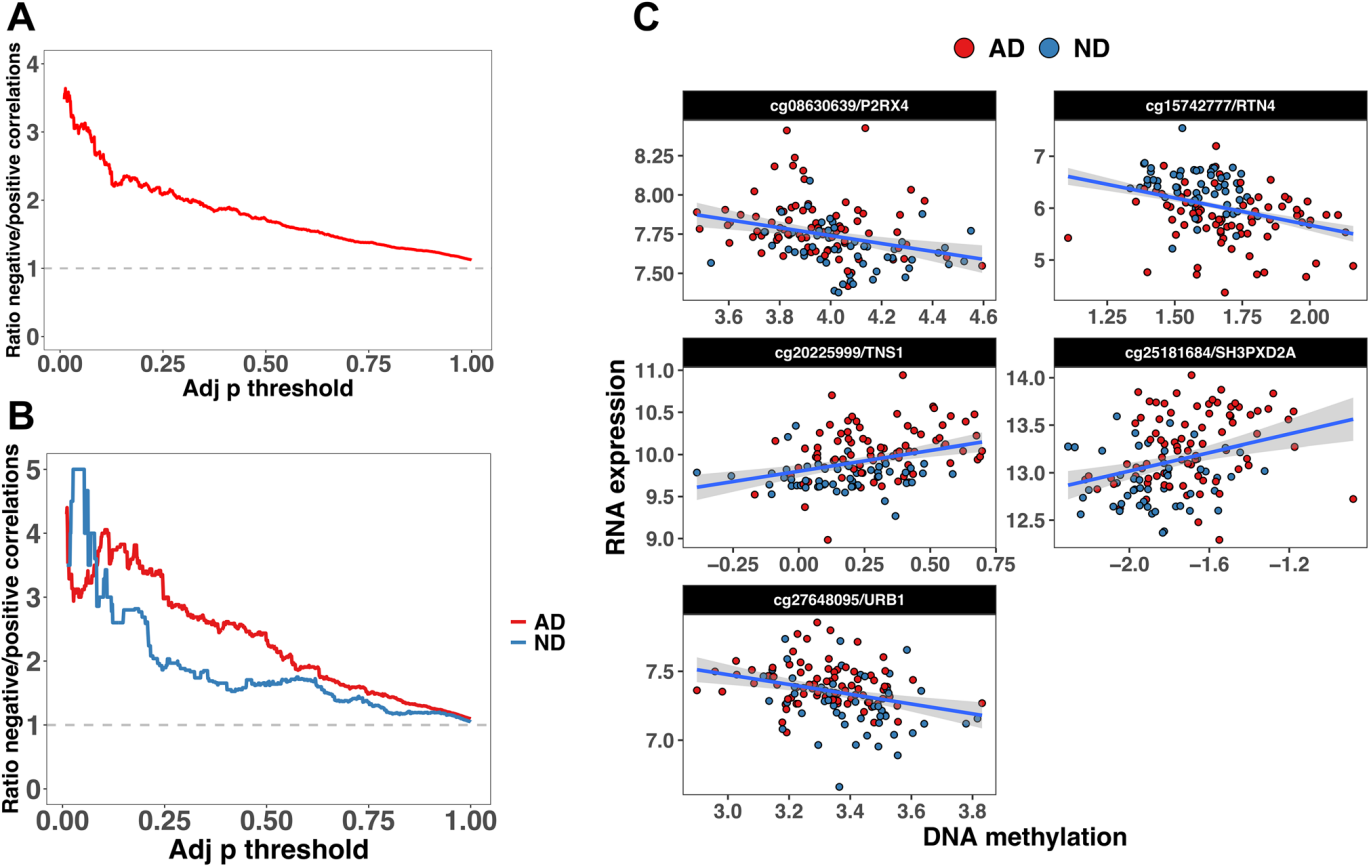
Ratio between negative and positive correlations as a function of the adj p cutoff in the total sample (**A**), and in AD and ND separately (**B**). **C** CpG/mRNA pairs significantly correlated (total sample: AD + ND) and differentially methylated and expressed

To assess the overall relationship between RNA expression and DNA methylation, we ran a biweight correlation between CpG sites located ± 1500 bp from each gene, and the expression level in *cis,* obtaining a total of 196,445 CpG/genes combinations, including 12,372 genes. When considering the total cohort (AD + ND), we found 92,521 positive and 103,924 negative CpGs/RNA correlations. The larger proportion of negative correlated CpG/RNA pairs was consistent across a wide range of *p*-values cutoffs (Fig. 2a), decreasing proportionally with the cutoff increase.

Only a small proportion of correlations were statistically significant: 43 positively correlated and 133 negatively correlated (Supplementary Table 11). We summarized the results at the gene level, selecting genes including at least 5 CpGs (10,617 genes and 191,567 CpG sites). We computed the average adjusted *R*^*2*^ for each gene, obtaining a total average *R*^*2*^ of 0.0023 (range: − 0.0074 to 0.242). Genes with large *R*^*2*^ (*R*^*2*^ > 0.10) were: *NAPTR1*, *PAX8*, *ORL2L13*, *NLRP2*, *DNAJC15,* and *CES1.* These genes included CpGs/RNA pairs significantly correlated (larger points in the figure) and CpGs/RNA pairs not significantly correlated (smaller points). However, these genes did not show CpGs or RNAs differentially methylated or differentially expressed (Supplementary Fig. 11).

We look for AD-specific correlations by computing the correlation in AD and ND groups separately. Using all the CpGs, we confirmed the larger proportion of negative correlations compared to positive correlations. Additionally, we observed a larger proportion of negatively correlated sites in AD than in ND, consistent across a wide range of cutoffs (Fig. 2b). In AD, we found 70 CpG/mRNA pairs significantly correlated, 53 negatively correlated, and 17 positively correlated. In ND, we found only 12 significant correlations, including ten negatively correlated (Supplementary Table 12). A total of 64 correlations were AD-specific, whereas only three were ND-specific. Permutation analysis sampling the same number of AD donors as the ND (*n* = 53) and computing the correlation for the 68 significant CpGs/mRNA pairs in both AD and ND showed that the difference in the number of significant sites was due to the different sample size, and not to a real biological effect (*p* = 1.000). We compared the findings for AD and ND, intersecting the significant results, and found that 10.8% of CpG sites/mRNAs (*n* = 8) were significant in both AD and ND. Only 5.4% were significant only in ND, whereas 83.8% (*n* = 62) were significant only in AD. Among the genes significant in AD with more CpG sites, we found *NAPRT1* (*n* = 7), *PAX8* (*n* = 14) and *OR2L13* (*n* = 5) (Supplementary Table 12). We computed the average *R*^*2*^ by gene, detecting eight genes in the AD group with *R*^*2*^ > 0.10 (*NAPRT1*, *PAX8*, *OR2L13, NLRP2*, *TMEM173*, *DNAJC15*, *PDPR,* and *CES1),* and almost the same genes in ND (*NAPRT1*, *NLRP2*, *OR2L13*, *KLHDC8B*, *PAX8*, *TSTD1, PPM1M*, *CES1,* and *EFHB*). However, the correlation between AD and ND *R*^*2*^ was not significant (*p* = 0.018; *p* = 0.08).

In the total sample (AD + ND), we found five CpG/mRNA pairs significantly correlated and simultaneously differentially methylated and expressed in the comparison of AD vs ND (Fig. 2c and Supplementary Table 11). In the AD and ND separate analysis, we did not detect CpG/mRNA pairs simultaneously differentially methylated or expressed (Supplementary Table 12).

#### Overlap of Significant DMPs and DEGs

**Fig. 3:**
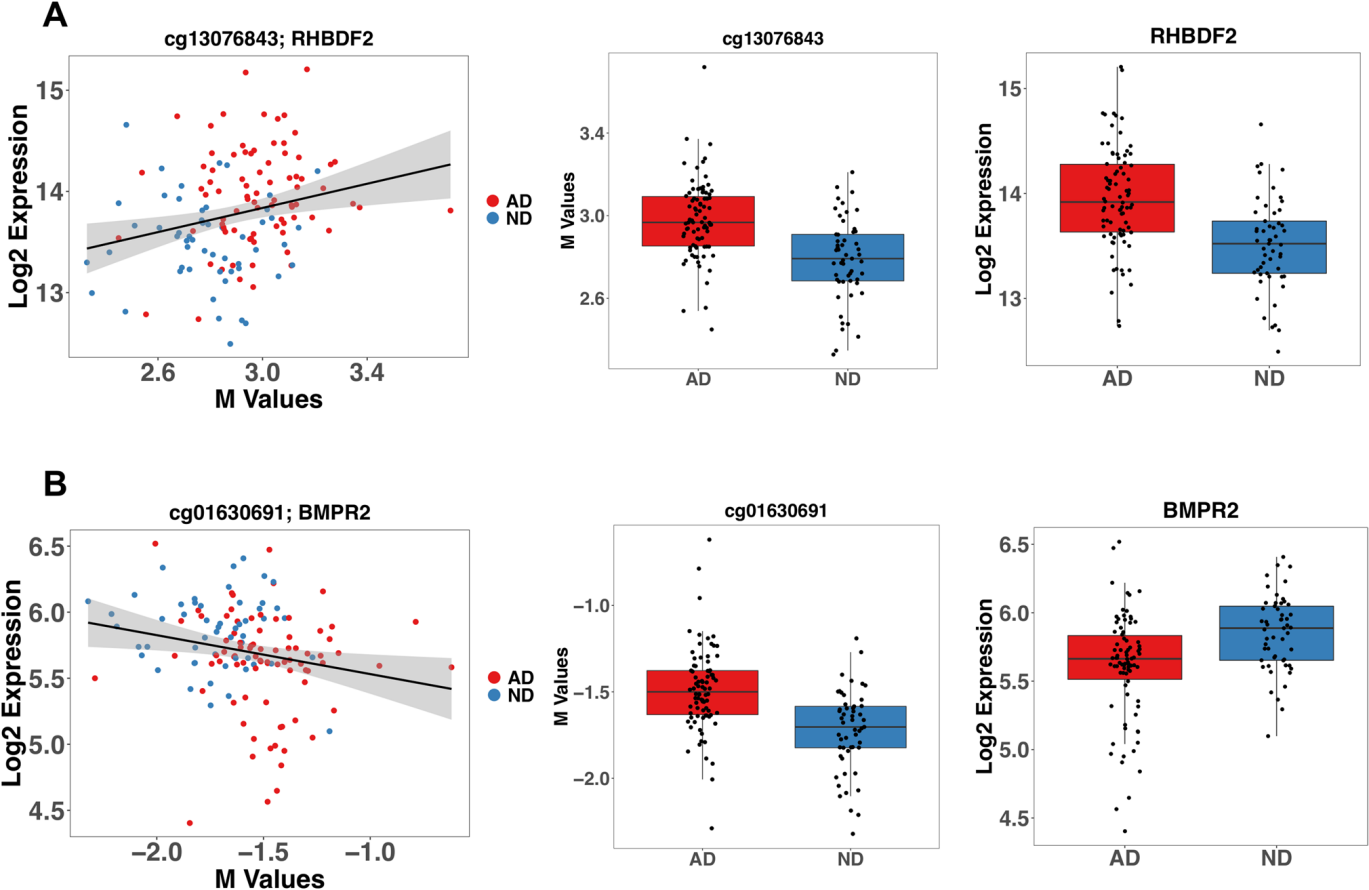
CpGs sites both differentially methylated and expressed

We integrated the DNA methylation data with the RNA expression profiling of an overlapping cohort from the same brain region (Piras et al. 2019a) for a total of 82 AD and 53 ND. We conducted differential RNA expression analysis on these 135 samples adjusting for age, sex, RIN, and PMI on 25,869 informative probes obtained after quality control filtering, detecting a total of 12,279 differentially expressed genes*.* We detected a small effect of the confounding factors, observing a strong and significant correlation with the differential results obtained with the raw model without adjusting for covariates (log2 FC: *ρ* = 0.966; *p* < 2.2E-16). We also observed a strong correlation between the RNA profiling results obtained using the DNA methylation matched samples (AD = 82; ND = 53) and the results obtained for the entire cohort (AD = 97; ND = 98) (log2 FC: *ρ* = 0.955; *p* < 2.2E-16) (Piras et al. 2019a). We conducted differential DNA methylation analysis for the 135 samples matched with the gene expression data obtaining 16 DMPs. Correlation between log2FC of the differential methylation analysis between the matched and total sample was very strong (log2 FC: *ρ* = 0.805; *p* < 2.2E-16). Then, we computed the differentially methylated regions in the same subsample, but we did not detect any differentially methylated region, possibly due to the decreased power (data not shown). We intersected the results from the DMP analysis with the differential expression analysis, detecting a total of 5 CpG sites differentially methylated located in genes differentially expressed: *RHBDF2*, *SLC25A26*, *ATP2B1*, *BMPR2*, and *PRDM16* (Supplementary Table 13). Because of the marginal significance, we checked all the CpG sites in the total cohort, and they all show a genome-wide significance. We decided to further explore the relationship between these CpG sites and their respective RNA by correlation analysis. After adjusting the correlation p-values for multiple testing accounting for this subset of comparisons (*n* = 5), we detected a positive significant correlation for cg13076843/*RHBDF2* (*r* = 0.248; *adj p* = 0.018) and a negative significant correlation for cg01630691/*BMPR2 (r* =  − *0.211; Adj p* = *0.035)* (Supplementary Table 13; Fig. 3A and B)*.* cg13076843 is located in exon 4 of *RHBDF2,* whereas cg01630691 is located in intron 4 (Fig 3).

### DIABLO Analysis

**Fig. 4:**
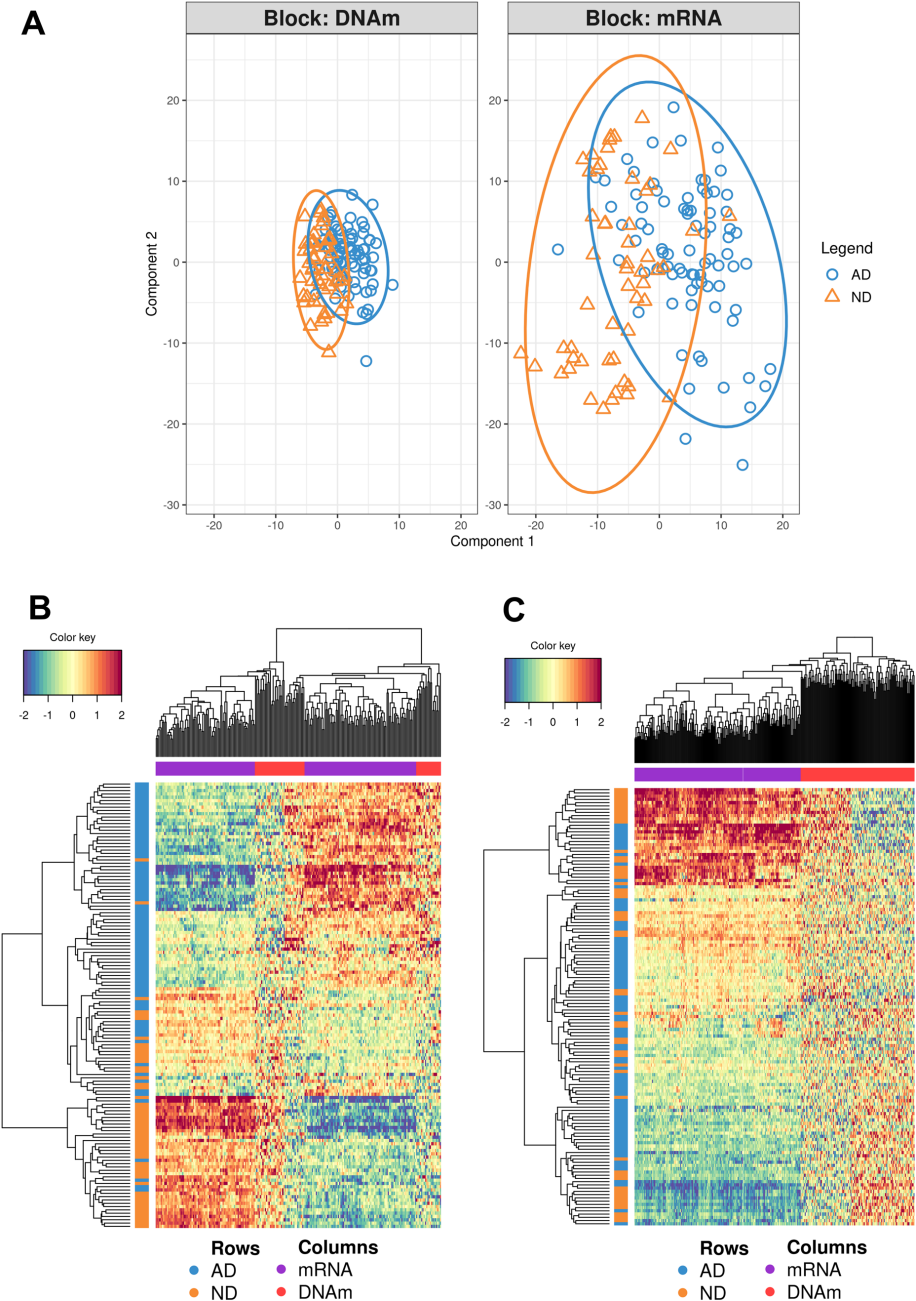
Discriminant power of the RNA and DNA methylation features used in the DIABLO analysis. **A** The first component, but not the second, shows a certain separation of AD and ND. **B** The heatmap generated using the first component shows a quite accurate classification of the AD and ND samples. **C** The heatmap generated using the second component shows an almost random distribution of the AD and ND samples

We tested the number of hyperparameters by cross-validation (5 folds × 20 repeats). We first estimated the number of optimal features testing from one to five components using the function *tune.spdla* (Supplementary Fig. 13)*.* Then, we ran the DIABLO model using the parameters obtained from this estimation, using the output to further tune the number of components with the function *perf* (Supplementary Fig. 14). The final model was run using two components with 170 and 190 features for RNA expression, and 60 and 130 features for DNA methylation. We obtained a clear separation between AD and ND for component 1 (AUC = 0.908), but little or no separation for component 2 (Fig. 4).

**Fig. 5:**
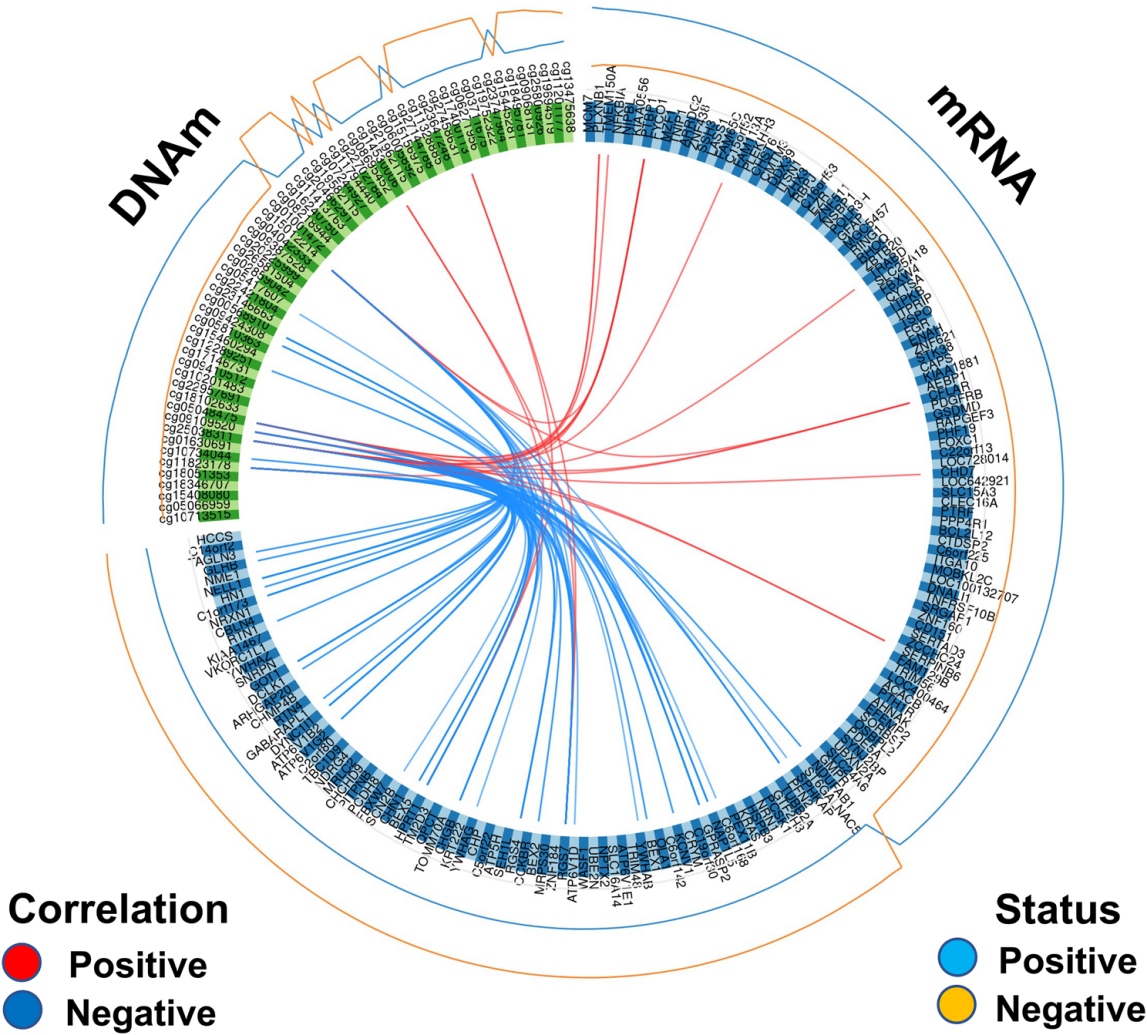
Circos plot representing the CpG sites strongly correlated with mRNA expression ( |*r*| ≥ 0.6) from principal component 1

We focused only on component 1, extracting the correlation matrix between CpG sites and mRNA filtering by *r* ≥ 0.60 (strong correlation), and visualizing the results in Fig. 5. We observed a total of 61 correlations between CpG sites and RNAs with *r* ≥ 0.60 (Supplementary Table 14). The contribution of each feature to component 1 is shown in Supplementary Fig. 15. The CpG site showing more RNA correlations (90.1% negatively correlated) was cg25038311 (*n* = 22), located in chr10:104,964,751, upstream the *NT5C2* gene. The site cg25038311 was significantly differentially methylated in the total sample (*Δβ* = *1.7%; adj p* = 1.6E-04, whereas *NT5C2* was not differentially expressed, underpinning some regulation mechanism in *trans* for cg25038311*.* The second site with more correlations was cg05048475 (*n* = 10), located in the body of the *SORT1* gene (intron 1), in a rich transcription factor region binding (SIN3A and MAX with the highest cluster score). cg05048475 was differentially methylated in the total sample (*Δβ* = 1.7%; adj p = 1.7E-02). The third CpG site was cg15012214 (*n* = 8), located in the *ARHGEF3* gene. The other CpG sites have five or less RNA correlated. Interestingly, cg11823178, located in the body of the *ANK1* gene, was negatively correlated with *CHGB* and *RGS4*. All the correlated genes (*n* = 23 unique genes) were differentially expressed, whereas only two CpG sites were differentially methylated in the matched sample (*n* = 135). However, they were all differentially methylated in the total sample (*n* = 290). We computed the correlation between the RNA expression of the genes correlated with the CpG and the RNA expression of the gene where the CpG was located. We found a significant correlation for *ARHGEF3* (adj-*p* ≥ 5.4E-15, *GPR56* (adj-*p* ≥ 2.9E-08), *GALNT2* (adj-*p* ≥ 5.0E-08), *CACNB2* (adj-*p* ≥ 2.02E-23), *RGMA* (adj-*p* ≥ 5.12E-17), and *MYO1C* (adj-*p* ≥ 3.2E-07). ARHGEF3/ cg1501221 had the largest number of significant correlations.

### FEM Analysis

**Fig. 6:**
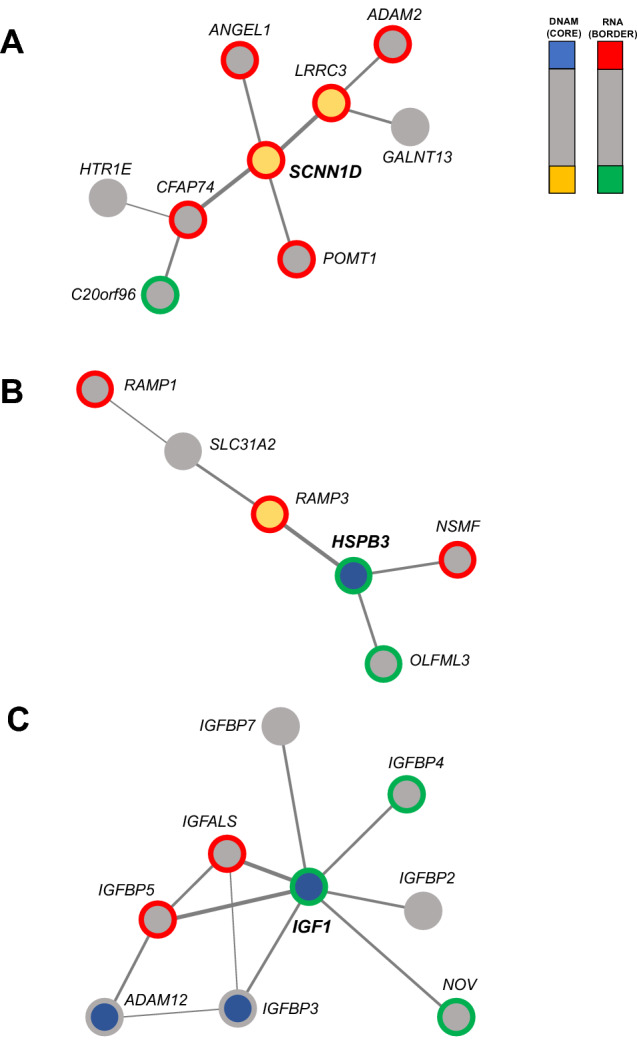
Significant protein–protein networks showing both differential methylation and expression. **A** Network showing significant enrichment for “O-linked glucosylation” including *SCNN1D* as a hub gene. **B** Network showing significant enrichment for “Amylin-receptor complex” including *HSPB3 *as a hub gene. **C** Network showing significant enrichment for “Regulation of insulin-like growth factor transport” including *IGF1* as a hub gene

Using the matched RNA expression and DNA methylation data, we identified a total of three significant epigenetic modules including the seed genes: *SCNN1D* (*p* = 3.9E-03), *HSPB3* (*p* = 4.3E-03), and *IGF1* (*p* = 6.1E-03), with 9, 6, and 9 genes, respectively (Supplementary Table 14). *SCNN1D* shows significant hypomethylation and upregulation in AD, and it is connected to *LRRC3*, showing the same pattern. Most of the other genes in the network show upregulation, but not any differential methylation (Fig. 6a). The genes in this network show enrichment in “O-linked glucosylation” (FDR = 2.4E-02; genes: *GALNT13* and *POMT1*). In the second network (Fig. 6b), the hub gene *HSPB3* showed differential hypermethylation and RNA deregulation in AD, and it is connected to *RAMP3*, showing an opposite pattern. The network is enriched for “Amylin-receptor complex” (FDR < 0.01; genes: *RAMP1* and *RAMP3*). Finally, *IGF1* shows hypermethylation and downregulation. The subnetwork is strongly enriched for “Regulation of insulin-like growth factor transport” (FDR = 1.16E-14; genes: *IGFBP5*, *IGF1*, *IGFALS*, *IGFBP2*, *IGFBP3*, *IGFBP4*) (Fig. 6c).

## Discussion

In this study, we reported the results of an integrated analysis between DNA methylation and RNA profiling of the middle temporal gyrus from 198 AD and 98 ND. RNA profiling data were reported in our previous studies, highlighting the presence of high gene expression dysregulation as well as co-expression modules associated with AD and related to RNA metabolism and mitochondria-associated membrane genes (Piras et al. 2019a, b). To the best of our knowledge, this is one of the largest EWAS conducted in AD postmortem brains, along with other studies published in the last years (De Jager et al. 2014; Lunnon et al. 2014; Semick et al. 2019). Furthermore, this study is focused on a brain region (middle temporal gyrus) not included in previous studies.

After strict quality controls and adjusting for confounding factors, we detected a total of 5246 DMPs sites and 832 DMRs. The top sites were cg11823178 and cg05066959, located in the *ANK1* gene and significantly associated also in the other three large EWAS. The two sites were significantly associated with neuropathological burden, ranking in the top positions by statistical significance. *ANK1* was differentially hypermethylated also in the DMR analysis, where the region included four sites. The role of *ANK1* in AD has been largely explored after the initial discovery in the early EWAS. Smith et al. (2019b) also detected the hydroxymethylation patterns in *ANK1*, demonstrating that the previous estimate of hypermethylation was underestimated by hydroxymethylation. Furthermore, hypermethylation in *ANK1* was detected also in other neurodegenerative diseases such as Huntington’s and Parkinson’s disease (Smith et al. 2019a). We did not find RNA differential expression of *ANK1* in our cohort. Mastroeni et al. (2017), using LCM in AD hippocampus, detected a significant upregulation in microglia but not in neurons or astrocytes, pointing out that the signal in RNA expression bulk tissue might not be detected due to the cell heterogeneity. However, in the AD scRNA study from Mathys et al. (2019) (dorsolateral prefrontal cortex), *ANK1* was not detected in microglia and was significantly downregulated only in excitatory neurons in the individual model (log_2_ FC =  − 0.248; adj *p* = 1.1E-18), but not in the mixed model. Finally, it was showed reduced expression of *Ank2* (ortholog of human *ANK1*) in *Drosophila* causes shortened lifespan, reduced locomotion, reduced memory, and reduced neuronal excitability (Higham et al. 2019).

In the differential methylation analysis, we detected a low effect size. The median *Δβ* for the DMPs (adj-*p* < 0.05) was 0.007 (0.7% in DNA methylation variation), with the largest value as 4.7%. Additionally, only 34.2% of CpGs had a variation equal to or larger than 1%. Previous studies on AD brains were able to detect larger effect sizes. For example, Semick et al. (2019), in their cross-brain analysis (four brain regions), detected a median Δβ of 3.98%. In the study of Lunnon et al. (2014), the Δβ difference ranged from 1 to 5%. However, in the first case, the inclusion of different brain regions might have increased the variability, and in the second case, the larger *Δβ* might be related to the difference between donors with Braak Stage 0 and VI, and between 0 and IV. In our study, the control group included donors with Braak Stage from 0 to III, and the AD group included samples with Braak Stage from IV to VI. Indeed, when we computed the *Δβ* comparing Braak Stage VI versus Braak Stage I, we obtained a larger median value (median *Δβ%* = 1.2%; maximum 8.0%) for the significantly associated CpGs than in the differential methylation analysis with all samples. We did not filter for effect size (for instance, *Δβ* ≥ 1%), since a large number of small effect size genes can cumulatively play a role in molecular pathways functions, and they are worth to be reported. Additionally, several of CpG sites with small effect sizes were validated in the comparison datasets, and the % of validation was not correlated to the effect size, suggesting that they might not be false positives.

We investigated the general relationship between RNA expression and DNA methylation using the matched cohort, detecting in both the whole sample (AD + ND) and in the single groups that a larger proportion of CpGs/RNA in *cis* negatively correlated. However, only a small proportion was statistically significant. This is not surprising, considering the complexity of the mechanisms regulating gene expression, not limited to DNA methylation. We detected a higher proportion of sites significantly correlated with AD than ND. However, permutation analysis showed that was only an effect of the sample size, due to the limited power of the ND cohort.

Using the DIABLO analysis, we found a strong negative correlation (*r* <  − 0.60) of cg11823178, located in the body of *ANK1,* with the RNA expression of CHGB and RGS4. The correlation of the other differentially methylated CpG sites located in *ANK1* was a little below the cutoff we used, showing *r* =  − 0.58. The two CpG sites in *ANK1* highly correlated with the expression of *CHGB* and *RGS4* are located in an enhancer element (GH08J041659), but the knowns regulatory interactions have been reported only in *cis* for *ANK1* and not for other genes in *trans*. The RNA expression of *ANK1* was not correlated with the RNA expression of *CHGB* and *RSG4,* so the CpG site in *ANK1* might exert some regulation independently of *ANK1* RNA levels. *CHGB* and *RGS4* in our previous study were included in the same co-expression network, associated with mitochondrial and organelle genes. *ANK1* was not included in the same coexpression module (perhaps because of the low variance), but we can speculate about a cell-specific effect not detectable in bulk tissue RNA sequencing. Our data, with the new association in the middle temporal gyrus, confirmed the key role of hypermethylation of *ANK1* in AD.

Other CpG sites highly differentially methylated and previously identified were located, among others, in *MYO1C* (cg05417607), *RHBDF2* (cg13076843, cg05810363), and *PCNT* (cg23449541). These three genes were differentially methylated also at the region level, with high significance for *RHBDF2* (log2 FC: 0.006; adj *p* = 1.94E-06; *n* = 6 sites), *MYO1C* (log2 FC: − 0.010; adj *p* = 1.08E-03; *n* = 2 sites) and marginal significance for *PCNT* (average M: adj *p* = 0.045; *n* = 5 sites). Interestingly, we found a positive significant correlation of cg13076843 (located in the body of *RHBDF2)* with RNA expression. The correlation with the gene expression of cg13076843 was previously reported also in the dorsolateral prefrontal cortex (De Jager et al. 2014). The other newly associated gene correlated with gene expression was *BMPR2* (Bone Morphogenetic Protein Receptor Type 2) (cg01630691), involved in vascular homeostasis (Cai et al. 2012), and it has been associated with a dysregulation in signaling mediated by TGF-β in AD (Canchi et al. 2019). Among the new associations, top CpG sites were located in *GPR56 (cg09109520)*, *COMMD3* (cg10713515), and *FAM20C* (cg23782833), and they were differentially methylated also in the DMR analysis. *GPR56* is part of the large family of G protein-coupled receptors genes, and multiple pieces of evidence show the protein product implicated in the hydrolytic processing of APP, as well as interactions with β- and γ-secretase (Zhao et al. 2016).

To better explore the relationship between these CpG sites and the RNA expression, we calculated the correlations between the RNA levels of the genes where the CpG site was located and the genes that were correlated. We found significant correlations for several genes (*ARHGEF3*, *GPR56*, *GALNT2*, *CACNB2*, *RGMA*, and *MYO1C*). An explanation might be a regulatory mechanism of the CpG site with its associated gene in cis, which is functionally correlated and then coexpressed with the other genes. Indeed, cg15012214 was located in *ARHGEF3*, and its expression was significantly correlated with the eight CpG target genes. Interestingly, all of them were included in the same coexpression network (turquoise) associated with the mitochondrial membrane (Piras et al. 2019a).

FEM analysis showed three significant networks, all of them associated in some way with amyloid-β. The most interesting network shows *IGF1* (insulin-like growth factor I) as a hub gene (downregulated and hypermethylated), and it is strongly enriched for “regulation of insulin-like growth factor transport”. IGF-I signaling has been found altered in AD brains (Frölich et al. 1998; Moloney et al. 2010), with increased dysregulation associated with disease progression (Ostrowski et al. 2016). Carro et al. 2002). However, it is still not well known whether IGF-1 dysregulation is a causal factor or a consequence of the disease (Galle et al. 2019). Decreased IGF-1 signaling has been associated with increased amyloid-β deposition and the development of phosphorylated tau (Cheng et al. 2005; Ashpole et al. 2015). Accordingly, in our study, we found cg08806558 (located in the *IGF1* promoter) significantly correlated with both Braak Stage and amyloid plaques. It was suggested that IGF-1 reduced brain β-amyloid enhancing the clearance through carrier proteins like albumin and transthyretin. The Framingham Heart Study observed a correlation between low levels of serum IGF-I at baseline and increased dementia risk (Westwoo et al. 2014). However, the result was not replicated in the Caerphilly Prospective Study (Green et al. 2014) and from Galle et al. (2019).

The network showing *SCNND1* as the hub gene was enriched for “O-linked glucosylation”, a protein posttranslational modification relevant in AD (Schedin-Weiss et al. 2014). APP has several O-glycosylation sites (Halim et al. 2011), and the process is necessary for the proper APP transport, with mutation impairing the process and resulting in APP accumulation (Tomita et al. 1998). Furthermore, the O-Glycosylation is involved in APP processing (Liu et al. 2017). Finally, the third network (hub gene: *HSBP3*) was enriched for the “Amylin-receptor complex” (genes: *RAMP1* and *RAMP3*). Amylin is a pancreatic hormone involved in glycemic control and in energy balance that has received interest because of findings about both beneficial and pathological effects associated with AD (Mietlicki-Baase 2018). For instance, amylin can reduce the amyloid burden in the brain also improving cognitive symptoms of AD (Adler et al. 2014; Wang et al. 2017). However, other studies showed an association with neurotoxicity (Jhamandas and MacTavish 2012), and accumulation in the brain of AD patients with colocalization with Amyloid-beta (Jackson et al. 2013). *RAMP1* and *RAMP3* genes were demonstrated to be strictly associated with amylin, controlling amylin’s effects on energy balance and food intake in mice (Coester et al. 2020).

## Conclusions

In conclusion, our study shed light on the DNA methylation patterns of a brain region not explored yet in epigenomic studies. First, we confirmed CpG sites associated with AD in previous studies but in different brain regions, located in genes such as *ANK1*, *MYOC*, and *RHBDF2.* Additionally, we detected new associations (e.g., *GPR56*). Integrative analysis of DNA methylation and matched RNA profiling data suggested a modest level of correlation, according to the complexities in gene expression regulation. Finally, we highlighted a PPI subnetwork enriched for “Regulation of insulin-like growth factor transport, including genes simultaneously differentially methylated and expressed in AD.

## Supplementary Information

Below is the link to the electronic supplementary material.

Supplementary file1 (DOCX 5219 KB)

Supplementary file2 (XLSX 9159 KB)

## Data Availability

Enquiries about data availability should be directed to the authors.
