# Supplementary material for: Integrated DNA Methylation/RNA Profiling in Middle Temporal Gyrus of Alzheimer’s Disease
Source: Cell Mol Neurobiol. Author manuscript; Available in PMC 2024 Jul 1. (PMC10287777; doi:10.1007/s10571-022-01307-3)

**Integrated DNA methylation/RNA profiling in middle temporal gyrus of Alzheimer’s Disease**

Ignazio S. Piras^1^, Danielle Brokaw, Yinfei Kong^2^, Daniel J Weisenberger^3^, Jonida Krate^1,+^, Elaine Delvaux^5,^, Swapna Mahurkar^6^, Adam Blattler^3,7^, Kimberly D, Siegmund^10^, Lucia Sue^11^, Geidy E Serrano ^11^, Thomas G. Beach^11^, Peter W Laird^12^, Matthew J. Huentelman^1^, Paul D. Coleman^5^ ,*.

*^1^Neurogenomics Division, Translational Genomics Research Institute, Phoenix, AZ, 85004, US.*

*^2^Department of Information Systems and Decision Sciences, California State University Fullerton, Fullerton, CA, 92831, USA*

*^3^Department of Biochemistry and Molecular Biology, University of South California, Los Angeles, CA, 90033, US*

*^4^L.J. Roberts Center for Alzheimer's Research Banner Sun Health Research Institute, Sun City, AZ, 85351, US*

*^5^Biodesign Institute, Neurodegenerative Disease Research Center, Arizona State University, Tempe, AZ, 85287, US*

*^6^UCLA Division of Digestive Diseases, University of California, Los Angeles, CA, 90024, US*

*^7^Genetics Graduate Group, University of California, Davis, CA, 95616, US*

*^10^Department of Preventive Medicine, University of Southern California, Los Angeles, CA,* 90089-9175, US

*^11^Civin Laboratory of Neuropathology at Banner Sun Health Research Institute, Sun City, AZ, 85351, US*

*^12^Center for epigenetics, Van Andel Institute, Grand Rapids, MI, 49503, US*

*^+^ Current Institution: UnityPoint Clinic, Waterloo, IA*

**Supplementary Fig. 1.**

Density plots of the *β*-values before and after quantile normalization (AD = 194; ND = 96).

**
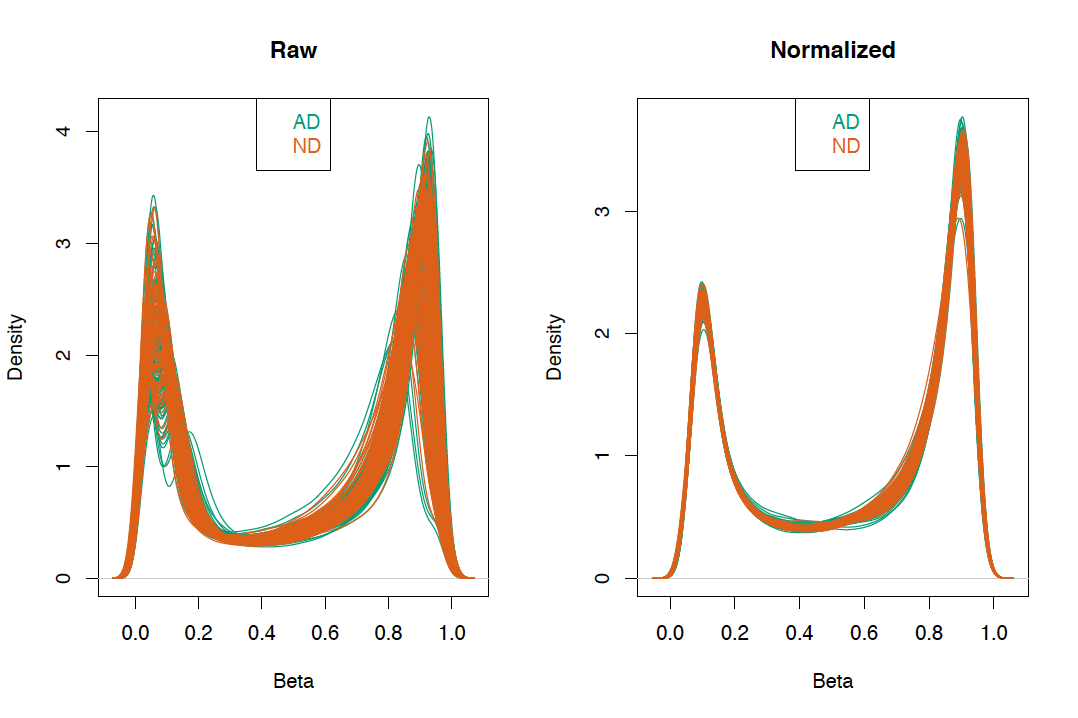
**

**Supplementary Fig. 2.**

MDS analysis (components 1 and 2) using the probes included after filtering steps (n = 381,974). Samples were colored by diagnosis (A), and plate (B)

**Supplementary Fig. 3.**

MDS analysis (components 1 and 2) using the probes included after filtering steps (n = 381,974) and after adjusting the M-values for confounding factors (age, sex, PMI, cell proportions, and plate). Samples were colored by diagnosis (A) and plate (B).

**Supplementary Fig. 4:**

1. Distribution of expired age (years) between AD (*n* = 194) and ND (*n* = 96). Average age (years) was 83.8 (range: 65 – 102) for AD, and 82.2 (range: 65 – 102) for ND. AD showed a larger average age than ND (t-test: *p* = 0.071).
2. Sex distribution between AD and ND. In the AD group, the prevalence of Males was lower than in ND. The distribution of sexes between AD and ND was significantly different (*p* = 4.4^-04^).

**Supplementary Fig 5.**

1. Distribution of PMI (hours) between AD and ND. The average and median PMI in AD were 3.26 and 2.83 (range: 1.33 – 45.00), and the average and median PMI in ND were 2.98 and 2.71 (range: 1.25 – 15.50). AD showed a larger average PMI than ND (Wilcoxon test: *p* = 0.247).
2. Distribution of PMI (hours) between AD and ND after removing the outlier included in the AD group. The average and median PMI in AD were 3.04 and 2.83 (range: 1.33 – 14.00), and the average and median PMI in ND were 2.98 and 2.71 (range: 1.25 – 15.50). AD showed a larger average PMI than ND (Wilcoxon test: *p* = 0.275).

**Supplementary Fig. 6**

1. Distribution of Braak Stage in AD and ND. The percentages shown are relative to each group according to each diagnosis group.
2. Distribution of Plaque density in AD and ND. The percentages shown are relative to each diagnosis group.

**Supplementary Fig. 7**

1. Distribution of the Brain Weight (BW) between AD (*n* = 194) and ND (*n* = 96). Average BW was 1057.4 (range: 670 – 1430) for AD, and 1215.7 (range: 890 – 1560) for ND. AD showed a very significantly lower BW than ND (t-test*: p* < 2.2^-16^).
2. Distribution of MMSE between AD and ND. The median MMSE in AD was 10 (range: 0 – 28), average MMSE in ND was 29 (range: 25 – 30). AD showed a very significantly lower MMSE than ND (Wilcoxon test: *p* < 2.2^-16^).

**Supplementary Fig. 8.**

Estimation of cell-type composition using a deconvolution algorithm based on a Dorso-lateral Prefrontal Cortex flow-sorted dataset. AD showed a significant lower proportion of neuronal cells (*β* = -0.037 ± 0.009; *p* = 3.2^-05^).

**
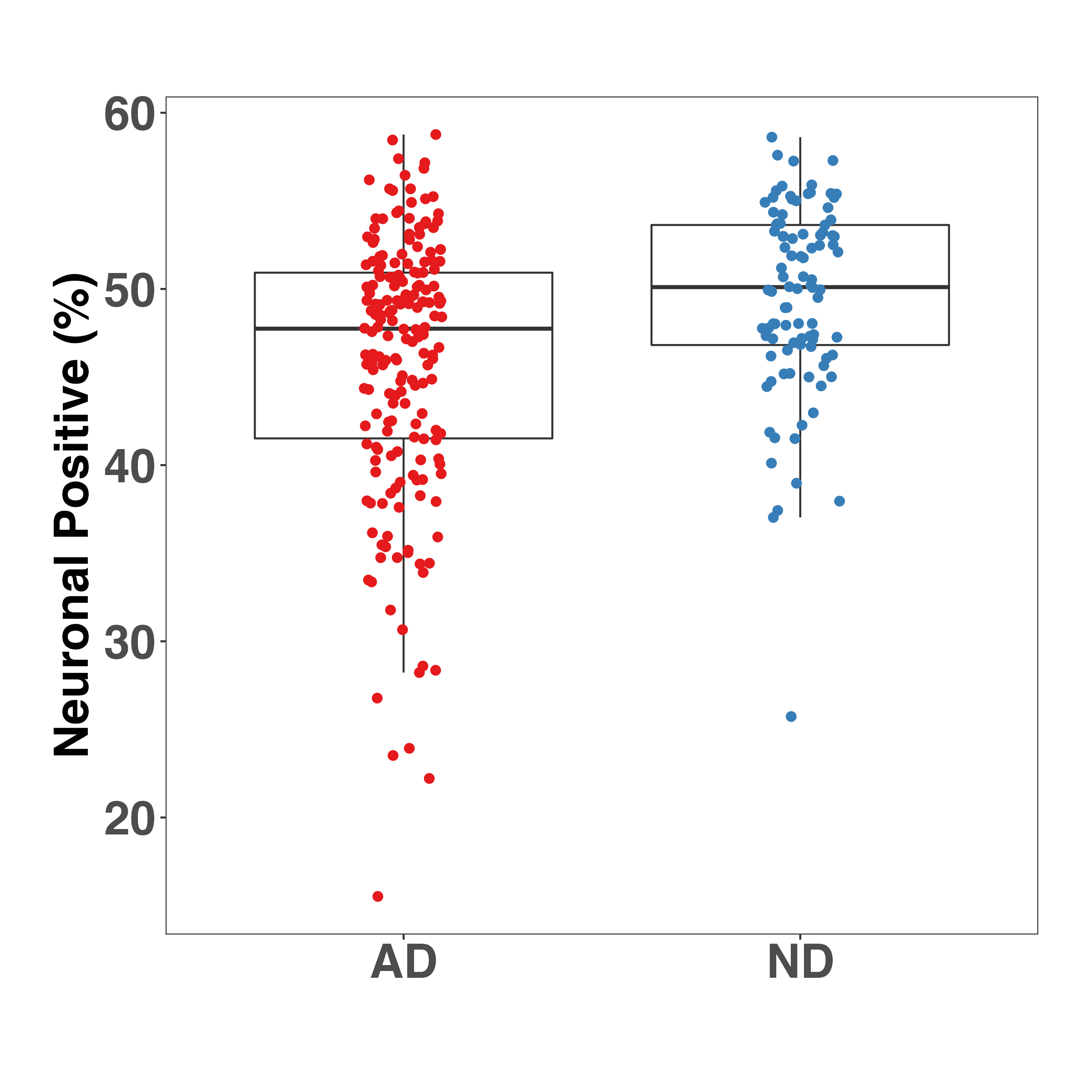
**

**Supplementary Fig. 9.**

1. We compared the distribution of genomic features density between the significant CpG sites (adj < 0.05) and the non-significant also by methylation status (adj *p* ≥ 0.05). We observed a significant difference between significant and non-significant CpG sites (*p* < 2.2-16). Among the significant CpG sites, we notice a significant reduction of CpG sites located on Island and an increase in Open Sea and Shelf regions (all p < 2.2^-16^). Only the difference in the distribution of CpG sites located in Shore regions was not statistically significant (*p* = 0.757).
2. We compared the distribution of CpGs in relation to genes. Overall, we found a significantly different distribution between significant and non-significant CpGs (*p* < 2.2^-16^). Additionally, the distribution was significantly different for each gene location. P-values ranged from 9.7^-03^ (5’UTR) to gene body (*p* < 2.2^-16^).

**Supplementary Fig. 10**. Number of CpG sites differentially methylated in our study validated in 1 or more datasets (percentage in X-axis).

**Supplementary Fig. 11**. Volcano plot representing -log10(*p*) as a function of bicorrelation coefficient for the genes with R^2^ > 0.10. Red and blue dots are CpG/mRNA pairs positively and negatively correlated, respectively. Larger dots show pairs significantly correlated (adj *p* < 0.05).

**Supplementary Fig. 12**. Results of the differential RNA expression analysis in the dataset, including 135 overlapping samples with the methylation study. Results were adjusted for age, sex, PMI, and RIN.

**Supplementary Fig. 13**. We tested the optimal number of features from 1 to 5 components for both RNA (top) and DNA methylation data (bottom).


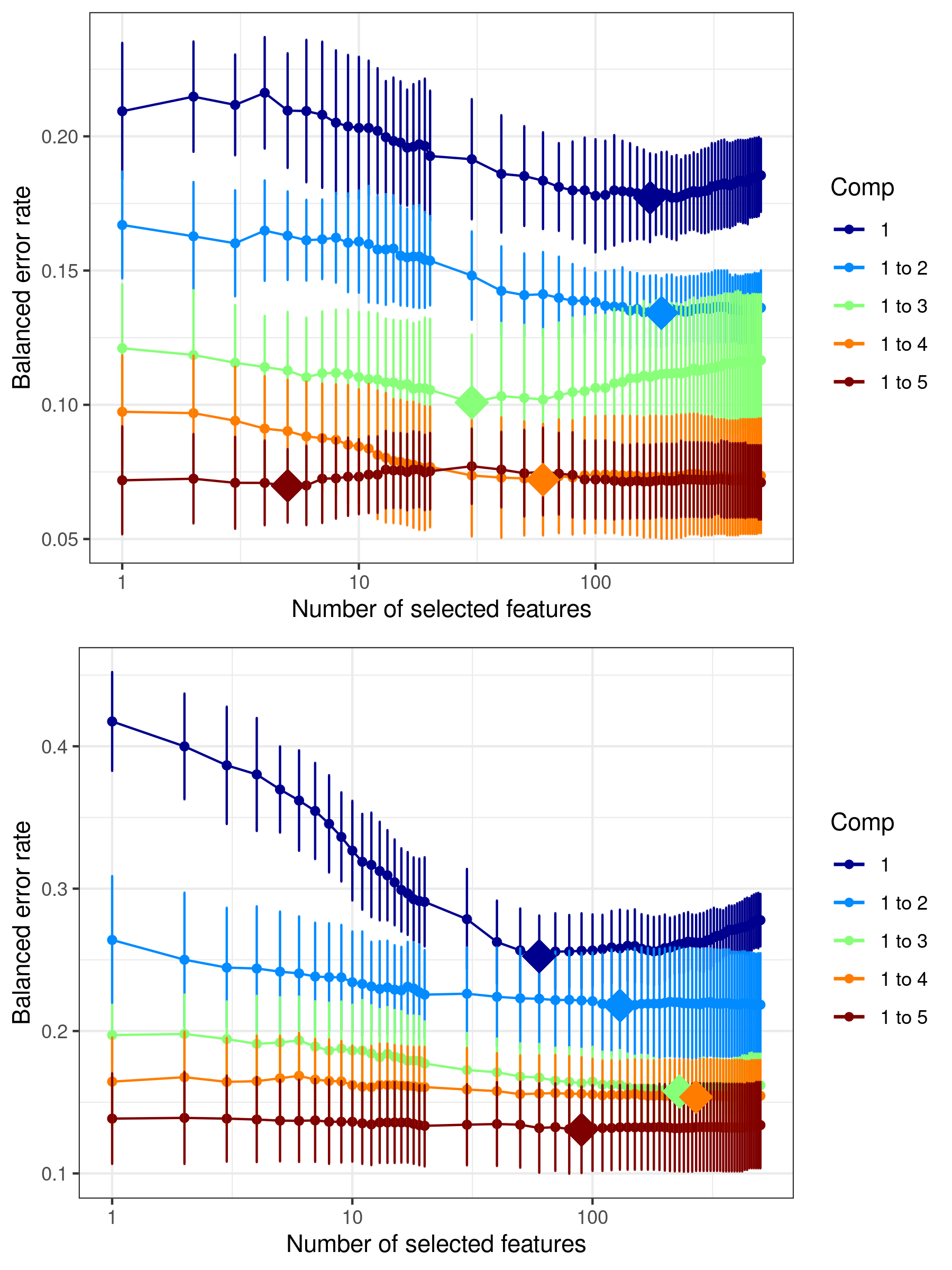


**Supplementary Fig. 14**. Estimation of the optimal number of components. Based on the error rate for both RNA expression and DNA methylation data estimated with three distance metrics, we decided to use two components to run the final model.


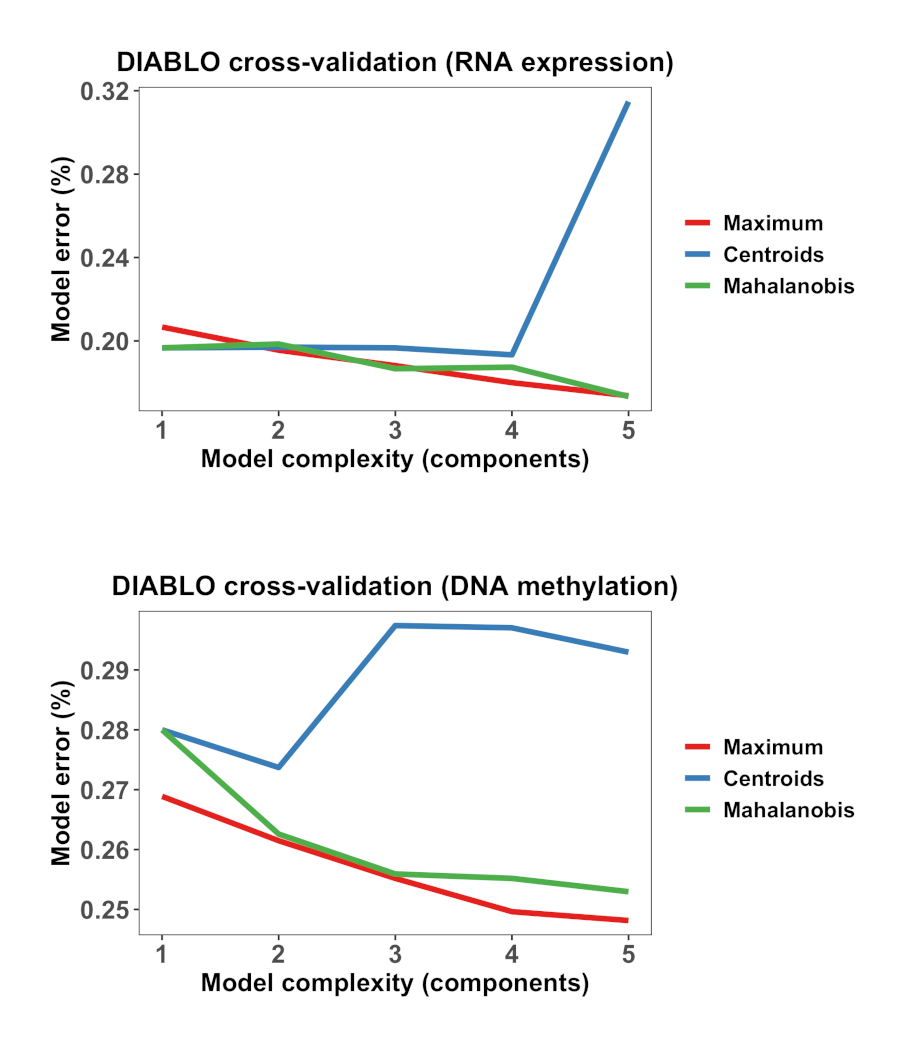


**Supplementary Fig. 15**. Contribution of each feature to component 1 in the DIABLO analysis.


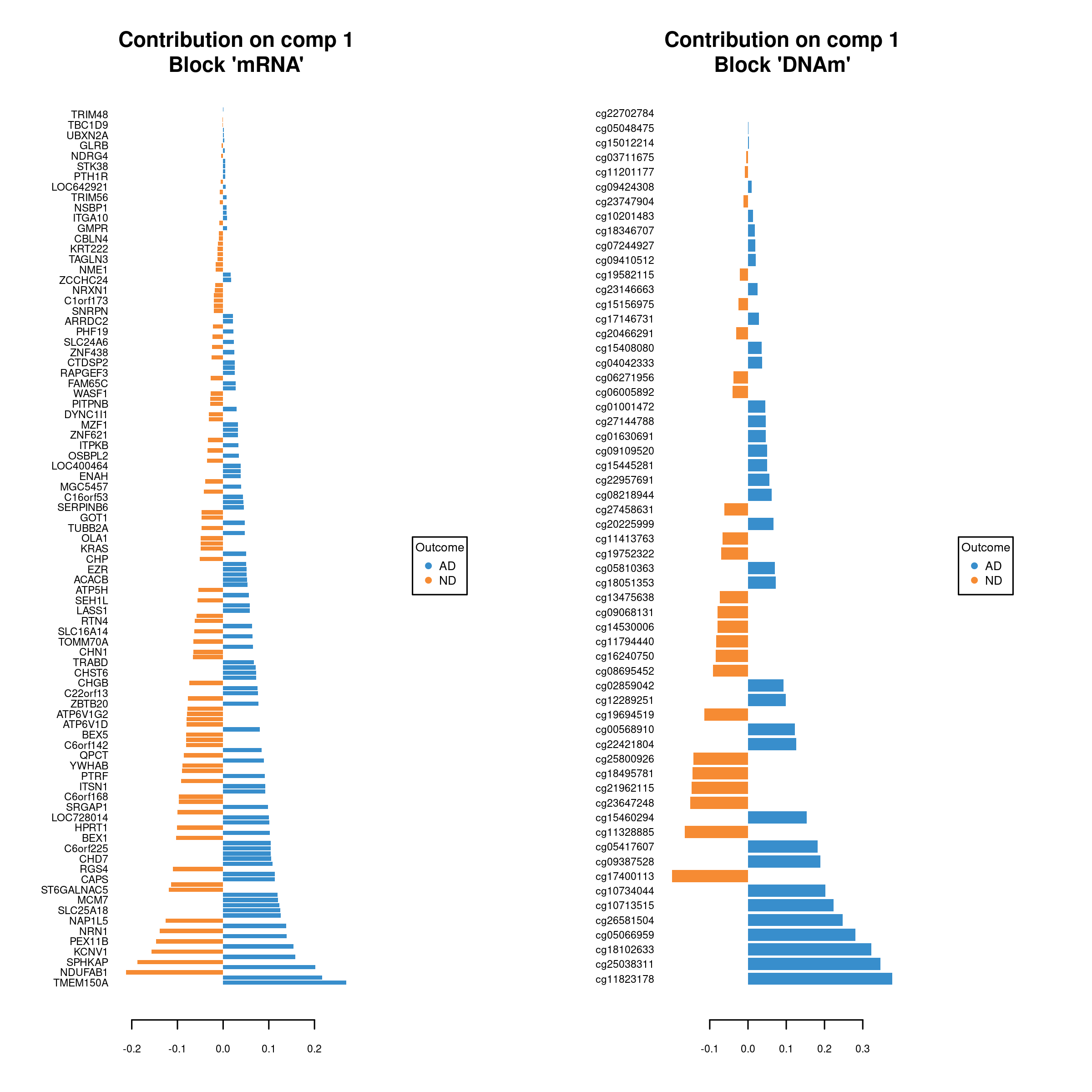

Supplement: Supplementary Results [file NIHMS1891990-supplement-Supplementary_Results.docx]
